# Transcriptional Profiling in Pathogenic and Non-Pathogenic SIV Infections Reveals Significant Distinctions in Kinetics and Tissue Compartmentalization

**DOI:** 10.1371/journal.ppat.1000296

**Published:** 2009-02-13

**Authors:** Sharon Lederer, David Favre, Kathie-Anne Walters, Sean Proll, Bittoo Kanwar, Zeljka Kasakow, Carole R. Baskin, Robert Palermo, Joseph M. McCune, Michael G. Katze

**Affiliations:** 1 Department of Microbiology, University of Washington, Seattle, Washington, United States of America; 2 Department of Medicine, Division of Experimental Medicine, University of California, San Francisco, California, United States of America; 3 Department of Pediatrics, Division of Gastroenterology, Hepatology, and Nutrition, University of California, San Francisco, California, United States of America; 4 Washington National Primate Research Center, University of Washington, Seattle, Washington, United States of America; University of Geneva, Switzerland

## Abstract

Simian immunodeficiency virus (SIV) infection leads to AIDS in experimentally infected macaques, whereas natural reservoir hosts exhibit limited disease and pathology. It is, however, unclear how natural hosts can sustain high viral loads, comparable to those observed in the pathogenic model, without developing severe disease. We performed transcriptional profiling on lymph node, blood, and colon samples from African green monkeys (natural host model) and Asian pigtailed macaques (pathogenic model) to directly compare gene expression patterns during acute pathogenic versus non-pathogenic SIV infection. The majority of gene expression changes that were unique to either model were detected in the lymph nodes at the time of peak viral load. Results suggest a shift toward cellular stress pathways and Th1 profiles during pathogenic infection, with strong and sustained type I and II interferon responses. In contrast, a strong type I interferon response was initially induced during non-pathogenic infection but resolved after peak viral load. The natural host also exhibited controlled Th1 profiles and better preservation of overall cell homeostasis. This study identified gene expression patterns that are specific to disease susceptibility, tissue compartmentalization, and infection duration. These patterns provide a unique view of how host responses differ depending upon lentiviral infection outcome.

## Author Summary

Simian immunodeficiency virus (SIV) does not cause disease in African green monkeys (a natural host for the virus), whereas experimentally infected Asian macaques (a non-natural host) develop a progressive disease that is similar to that which occurs in HIV-infected humans. Insight into how HIV causes disease and leads to development of AIDS may therefore be gained by comparing the response of natural and non-natural hosts to SIV infection. To this end, we examined changes that occurred in gene expression levels over time and in multiple tissues derived from African green monkeys and Asian macaques experimentally infected with SIV. Infection leads to host-specific gene expression patterns in lymph nodes, blood, and colon. The natural and non-natural hosts differed with respect to the timing, intensity, and duration of infection-induced gene expression changes associated with inflammation and response to stress.

## Introduction

Natural reservoir hosts of simian immunodeficiency virus (SIV) do not develop AIDS in response to infection and live a normal lifespan. This is in contrast to non-natural hosts, such as Asian pig-tailed macaques (PTs), which, when experimentally infected with SIV, develop AIDS in a similar manner to HIV-infected humans [1],[2]. Pathogenic SIV infection is characterized by high viral replication, loss of CD4+ T cells, high immune activation, T cell apoptosis, and ultimately, AIDS [3]–[5]. SIV-infected natural hosts, such as African green monkeys (AGMs), retain stable CD4+ T cell counts in peripheral blood even in the presence of viral loads (VLs) as high as those observed during pathogenic infection [6]–[8]. Given this dichotomy, insight into the mechanisms of HIV pathogenesis might be gained by comparing the transcriptional response of natural and non-natural hosts to SIV infection.

High levels of viral replication and the loss of CD4+ T cells correlate with faster disease progression in pathogenic SIV/HIV infection [9]–[11]. Diseased hosts also exhibit substantial loss of uninfected CD4+ and CD8+ T cells as a result of activation-induced cell death [12],[13]. It is thought that adaptive (T and B cell) antiviral immune responses may serve to control viral replication and spread [14],[15]. Yet, high levels of CD8+ T cell activation are also predictive of disease progression [16]–[18].

Cytotoxic T cell (CTL) responses are associated with increased induction of inflammatory cytokines, which in turn also correlate with pathogenesis. Conversely, control of immune activation in non-pathogenic infection may relate to anti-inflammatory cytokine profiles (reviewed in [19]). Dysregulation of the pro-inflammatory response has been reported in pathogenic lentiviral infections, often as a result of cytokine induction by viral proteins [20],[21]. Secretion of CC-chemokines has been associated with an HIV-induced proinflammatory cytokine profile [22],[23], and molecules such as interferon gamma (IFNγ) are up-regulated in lymphoid tissues of SIV-infected rhesus macaques [24],[25]. Although IFN responses are critical components of the innate immune response to viral pathogens, type 1 interferon expression is elevated in the acute phase of pathogenic SIV infection of non-human primates (NHPs) and persists in lymphoid tissue but fails to control viral replication [25],[26]. Additionally, a strong innate response to SIV often includes induction of genes recognizing pathogen-associated molecular patterns (PAMPs). It has been proposed that increased expression of inflammatory mediators may be a result of general immune activation caused by Toll-like receptor (TLR) induction by bacterial products escaping the gut [27]. Such bacterial translocation may be a result of epithelial damage from a sustained pro-inflammatory environment in the gut mucosa [28].

Increased levels of pro-inflammatory cytokines in HIV and pathogenic SIV infections have also been associated with T cell apoptosis via tumor necrosis factor (TNF)/FAS death receptor signaling pathways [29]–[31]. Lower levels of immune activation have been correlated with decreased FAS-induced cell death in HIV patients undergoing retroviral therapy [32], and levels of immune activation and T cell apoptosis are low in natural SIV hosts with non-pathogenic infections [8],[33],[34]. Conversely, bystander activation induced cell death is associated with higher levels of T cell activation [12],[13],[35]. Excessive early T cell apoptosis has been examined in pathogenic SIV models [36],[37] and has been proposed as a mechanism for pathogenesis. A positive correlation between primary acute-phase apoptotic levels in peripheral lymphoid tissue and disease progression in rhesus macaques has been demonstrated [36]. In addition to sustained immune activation, T cell apoptosis in pathogenic models may be linked to dysregulation of the cell cycle, as perturbations have been observed in lymph nodes of SIV-infected macaques but not in naturally-infected sooty mangabeys [38].

The mechanisms by which inflammatory responses, T cell activation, and apoptosis are induced, sustained, or suppressed differentially between pathogenic and non-pathogenic infections remain unclear. To delineate such mechanisms, we performed a longitudinal genomic analysis comparing the transcriptional responses of natural (AGM) and non-natural (PT) hosts in the setting of acute SIV infection with the same primary isolate (SIVagm.sab92018). For the microarray experiments, each animal was its own genetically matched control for assessing gene expression changes, with multiple animals for each experimental condition, enabling us to perform statistical tests to identify those gene expression changes that distinguish pathogenic from non-pathogenic SIV infection. Our observations revealed that SIV induced host-specific global gene expression patterns in lymph node (LN), blood, and colon. In more than one respect, this study provides a unique view of lentiviral infection in vivo. First, we now have a global perspective of the host transcriptional response to SIV in pathogenic and non-pathogenic models. Second, several tissues that are known to be affected by lentiviruses were examined at multiple time points, thus providing significant insight into the dynamics of the acute phase of infection. Our analysis of these tissues separately and over time has revealed new insights into the kinetics and compartmentalization of interactions between virus and host.

## Results

We examined the global gene expression changes that occur during the acute phase of pathogenic and non-pathogenic SIV infection in NHPs. Four PT macaques (*Macaca nemestrina*) and four AGMs (*Chlorocebus sabaeus*) were each infected intravenously with 600 TCID_50_ of SIVagm.Sab92018 [6]. The course of infection was followed for 6 weeks, and LN, blood, and colon biopsies were collected from each animal at baseline (day −14), at peak viremia (day 10), and at the conclusion of the experiment (days 45 or 49, referred to as “day 45+”). For each microarray experiment, mRNA levels from individual animals were compared to those at baseline (day −14) for the same animal. Extensive virologic, immunologic, and histologic characterizations were also performed with these and additional samples, and are described in part in a separate manuscript [39]. SIV viremia was detectable in PTs and AGMs by day 3 post-infection and reached high peak levels (exceeding 10^8^ RNA copies/ml) in the blood by day 10, after which it declined and remained stable at high viral “set-points” (Figure S1A). In the PT relative to the AGM, there were increased viral loads that reached statistical significance (*P*<0.02) in peripheral blood mononuclear cells (PBMCs) (at day 3), colon (at day 10), and lymph node (at day 45+). Otherwise, cellular RNA viral load ranges were similar between the two species. After infection, CD4+ T cell counts and frequencies remained stable in the blood and lymph nodes of AGMs but were rapidly depleted in PTs (Figure S1B). By contrast, and as recently reported [40],[41], CD4^+^ T cell depletion in the colon was similar in magnitude and kinetics in both species, with the frequency declining to low levels by day 10.

**Figure 1:**
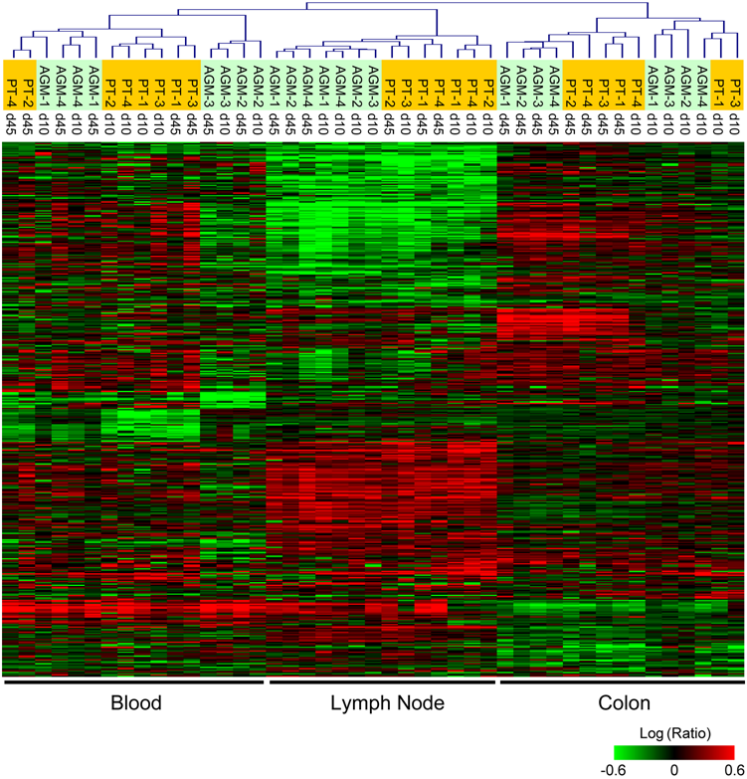
Global gene expression analysis in blood, lymph node, and colon during acute SIV infection of African green monkeys and Pigtails. Two-dimensional hierarchical clustering was done using Resolver System software with an agglomerative algorithm, average link heuristic criteria, and Cosine correlation metric. Each column represents gene expression data from an individual experiment, including all PTs (orange highlight) and AGMs (green highlight) at days 10 or 45+ post-infection, and all tissues. The cluster represents genes that showed ≥2-fold change (*P*≤0.05) in at least two experiments. Genes in red are up-regulated, and genes in green are down-regulated, as compared to pre-infection levels for each animal.

The overall effect on host gene expression during the first weeks of SIV infection was significant. As shown in Figure 1, the expression levels of 9,470 genes on the array changed at least 2 fold in a minimum of 2 experiments, relative to pre-infection levels (*P*≤0.05). With unsupervised clustering, expression patterns of each tissue were distinct, with the highest levels of post-infection induction in LN, where there was a clear separation of PTs from AGMs. Genes differentially expressed in blood clustered closest to the LNs and both tissues shared many patterns, to varying degrees. Expression patterns in colon were distinct from all other samples, and samples separated based on time post-infection, rather than by species.

### Overall gene expression changes in pathogenic and non-pathogenic SIV infection

**Figure 2:**
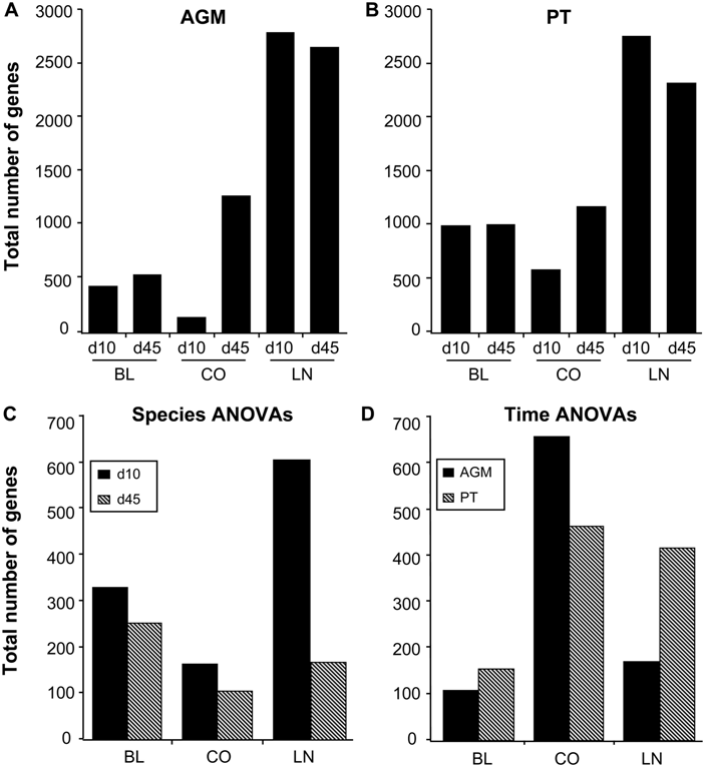
Quantitative analysis of total gene expression changes in blood (BL), colon (CO), and lymph node (LN) biopsies during acute SIV infection. Total number of differentially-expressed genes (≥2-fold change, *P*≤0.05) in AGMs (A) and PTs (B) for each tissue and time-point. Numbers are generated from in-silico pools of four animals/species/time point. (C) Numbers of genes identified by ANOVA (*P*≤0.01) distinguishing AGMs from PTs at day 10 or day 45+ in each tissue and species (D). Numbers of genes identified by ANOVA (*P*≤0.01) distinguishing day 10 from day 45+. Genes depicted in C and D were 2-fold regulated (*P*≤0.05) in at least 2 of 8 animals.

A quantitative depiction of differential gene expression is shown in Figure 2A and 2B. For this and all ensuing analysis, data points for four animals per species/tissue/timepoint were used (excluding AGM-3 and PT-3 at day 45+ in LNs, and PT-2 in colon at day 10, due to sampling errors). Overall, the greatest differential regulation of genes occurred in the LNs of infected animals, and the total number of differentially expressed genes was comparable between AGMs and PTs. The number of differentially regulated genes in the peripheral blood was relatively consistent between day 10 and 45+, though more changes overall were observed in PTs as compared to AGMs. In the colon of both species, the number of differentially expressed genes was greater at day 45+ (a time when viral set point had been established) than at day 10 (peak VL).

To identify genes whose expression was statistically different between the two species (and which therefore might be related to disease outcome), we performed error-weighted statistical tests (one-way ANOVA) on data collected from all animals at each time point, directly comparing AGM to PT for each tissue. Figure 2C is a summary of the total number of genes that were significantly different by species. Despite having comparable numbers of differentially regulated genes at day 10 in LNs, there were over 600 genes expressed at significantly different levels in the AGMs vs. PTs at this time. In all tissues, it was evident that the most gene expression changes distinguishing species were found at viral peak (day 10), with fewer distinguishing expression profiles at day 45+. In addition to comparing the species at each time-point by species ANOVA, we examined kinetics by comparisons of total gene expression changes between peak VL (day 10) and viral set-points (day 45+) in each species by time ANOVA (Figure 2D). Significantly more time-dependent changes occurred in the colon of both species than in blood or LN. These results suggest that events which occur during the very acute phase of infection play a key role in disease outcome.

### Evidence for stress responses and apoptosis during early SIV infection in the lymph nodes

**Figure 3:**
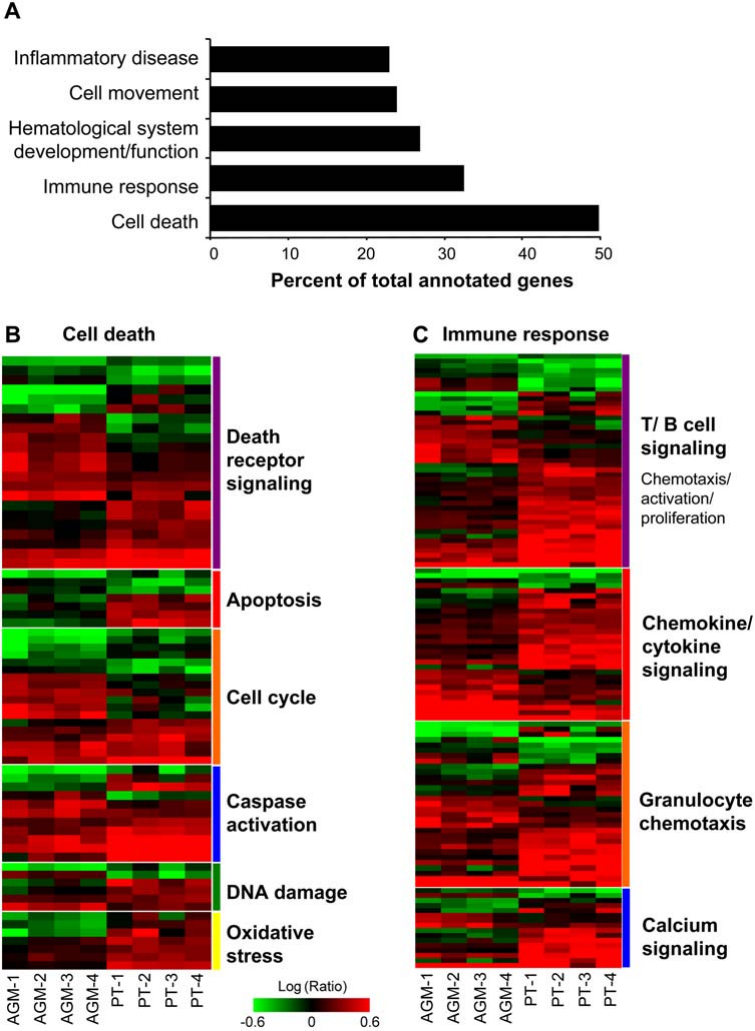
Gene ontology analysis of genes that statistically distinguish AGMs from PTs in lymph nodes at viral peak. (A) Gene annotation was performed using Ingenuity Pathway Analysis. Bars represent percent of genes in each category based on total numbers of annotated ANOVA set genes. (B) Expression profiles of 124 cell death-related genes from LN day 10 species ANOVA. (C) Expression profiles of 84 immune-response related genes from LN day 10 species ANOVA. Each column represents gene expression data from an individual animal with green and red colors showing decreased or increased levels of mRNA expression relative to d-14, respectively. Gene names for B and C are listed in Tables S1 and S2, respectively.

As most SIV-specific gene expression regulation was found in lymph nodes, we focused much of our subsequent analysis on this tissue. Based on one-way species ANOVA comparing PTs to AGMs, we identified 618 genes whose expression levels at day 10 were significantly different between the two species (Figure 2C). Gene ontology analysis revealed that the majority of these genes were associated with either immune responses or cell death (Figure 3A), with redundancies in the categories of hematological system development, cell movement, and inflammatory disease. Because the top categories are very broad, genes were further subdivided based on available annotations for additional insight into possible differences in cell processes.

Genes associated with cell death (shown in Figure 3B and Table S1) included those mediating death receptor signaling, apoptosis, cell cycle arrest and progression, caspase activation, DNA damage response, or oxidative stress. Although there were numerous genes associated with death receptor signaling, there did not appear to be a species bias towards pro- or anti- FAS-mediated apoptosis. Genes more highly induced in AGMs included UNC5B and STK11 (known to play a role in FAS-mediated apoptosis and associated with p53 signaling) and TRADD and IER3, which are positive or negative regulators of the FAS pathway, respectively. Genes that were more highly induced in PTs have functions more pertinent to the negative regulation of death receptor signaling (e.g., ASAH1, BTK, and CFLAR) and of apoptosis (e.g., IDE, CAMK2D, and LGALS3). Genes associated with oxidative stress and DNA damage (e.g., SOD2, DLST, GLRX, RRM2, YWHAE, and RAD51L3) also showed higher levels of induction in PTs. Collectively, these patterns are consistent with the possibility that oxidative stress is more prominent in pathogenic infection, prompting the induction of cellular genes that serve a protective role against oxidative stress, DNA damage, and apoptosis. Cell death genes related to the cell cycle were also differentially regulated between species. In general, a greater proportion of these genes were induced in AGMs (e.g., AXL, CDN2D, and MYCN), including those known to be responsible for both progression and arrest of the cell cycle.

### Gene expression associated with Th1 response, inflammation, and cytotoxic T lymphocyte (CTL) signaling in lymph nodes of non-natural hosts

Immune response genes that significantly distinguished the two species at peak VL (day 10) in lymph nodes consisted mainly of those associated with T and B cell signaling and with chemotaxis of immune cells (Figure 3C, Table S2). Compared to AGMs, significantly more genes were highly induced in PTs and they exhibited a trend towards Th1 differentiation and proliferation, cytotoxic T cell activity, and IFNγ signalling. These genes included inflammatory mediators (e.g., CXCL9 and 10, CCL3, CTSC, and CCR2) as well as genes necessary for antigen presentation or CTL responses (e.g., CD86, CD84, CD8a, GZMA, GZMB, CLEC4A and KLRC1). Although not identified by the ANOVA, PTs displayed a trend towards higher expression of additional genes for antigen-presentation (e.g., CCL17, CD80, GBP3, and ICOS), dendritic cell recruitment/maturation (e.g., ADAMDEC1, CCR7, ISGF6, and LAMP3), and CTL responses (e.g., CD2, CCL20, CLEC7A, and SRGN). These patterns are consistent with other studies showing that pathogenic infections can be associated with robust Th1 responses [34],[42],[43].

In PTs, significantly higher expression of T cell chemokine genes from the day 10 species ANOVA was consistent with a generally higher level of immune activation in this species, as evidenced by the presence of significantly more CD8+ T cell activation in PT LNs (by Ki67 levels) at both day 10 and day 45+ [39]. High induction of pattern-recognition receptors (e.g., CLEC7A, TLR2, and TL8) in PTs also suggest a strong innate immune response. Many genes regulating neutrophil chemotaxis and degranulation (e.g., LY75, LYZ, FCGR3B, CSF2BA, CEBPA, and B4GALT1) were only induced in PTs. A greater number of genes associated with calcium signaling was also induced in PTs, possibly reflecting the use of calcium-signaling pathways by broadly induced chemokines. Interestingly, the HIV/SIV co-receptor CCR5 was induced in all animals but at significantly higher levels in PTs than AGMs, in support of previous observations [44] and with a proposed model of restricted CCR5 expression in natural SIV hosts [19].

**Figure 4:**
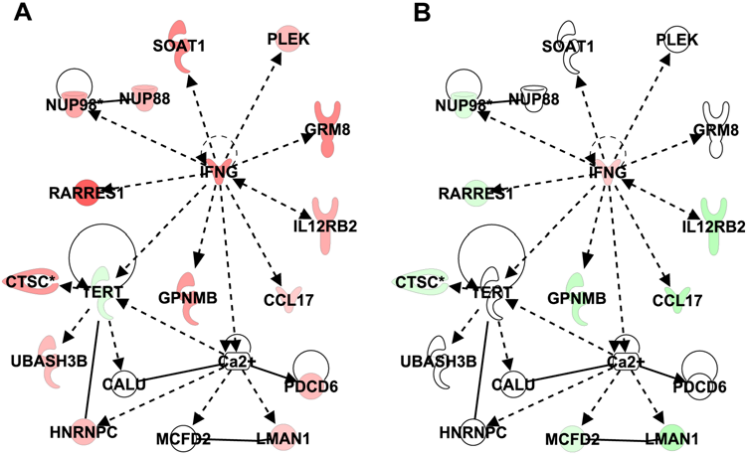
Expression of genes associated with IFN-γ signaling in lymph node of PT (A) and AGM (B) at day 10. Ingenuity network analysis showing genes associated with IFNγ signaling overlaid with expression data from in-silico pooled samples of PT LNs (A) or AGM LNs (B) at day 10. Genes shown in red and green represent increased or decreased expression, respectively, relative to pre-infection levels.

From the above LN d10 ANOVA, a subset of 137 genes showed increased expression only in PTs but decreased expression or no change in AGMs (Table S3). These genes encode mediators of inflammation, apoptosis, proliferation, and IFNγ signaling. Gene ontology analysis of this subset identified a significant network linked to IFNγ signaling and included genes that positively regulate or are regulated by IFN-γ (e.g., IL12RB2, RARRES1, GPNMB and NUP98) (Figure 4A). Others (e.g., CALU, HNRNPC, LMAN1, and PDCD6) are linked to calcium signaling, consistent with the data shown in Figure 3C. As genes in this network were down-regulated in AGMs (Figure 4B), these observations underscore the premise that T cell proliferation and strong Th1 responses are more specific to pathogenic than nonpathogenic SIV infection and, reciprocally, suggest an important role for the regulation of proliferation and inflammation in early stages of non-pathogenic SIV infection.

### Specific induction of genes associated with hematolymphoid system regeneration and resolution of Th1 signaling in AGMs

In contrast to PTs, highly-expressed genes in AGMs (day 10 species ANOVA, Figure 3) were associated with the control of an inflammatory/Th1 response and the development of hematopoietic progenitors. Notably, the AGMs showed up-regulation of the cytokine IL10, a potent anti-inflammatory mediator and negative regulator of Th1 cytokine production, as well as NLRP3, which is associated with prevention of inappropriate inflammatory responses [45]. This suggests a more active control of the inflammatory response in AGMs. CCL25, a B-cell chemokine and promoter of IgA production, was also induced at higher levels in AGMs. Genes associated with mast cell accumulation and degranulation included COL17A1, CCL2, and LTC4S [46]. Many additional genes induced in AGMs, or down-regulated in PTs, were associated with eosinophil migration or myeloid chemotaxis and growth (e.g., CCL2, IL10, LTC4S, CCL11, MADCAM1, and PLA2G6), or the development of hematopoietic progenitors (e.g., THY1/CD90, CD33, and CD34). Genes associated with the proliferation or differentiation of bone marrow cells (e.g., RARA) and of gamma delta T cells (e.g., JAG2) were also specifically induced in AGMs. Deficiencies in these functions have already been associated with disruption of homeostasis in pathogenic HIV/SIV infection [47],[48]. These gene expression patterns are consistent with the possibility that the AGM may be better poised than the PT to sustain repopulation of the peripheral hematolymphoid system after SIV infection [4].

A key to uncovering AIDS pathogenesis could lie in discerning what is unique to the response of natural hosts to SIV infection. From the LN d10 ANOVA, 108 genes were identified as significantly induced only in AGMs (Table S4). The functions of these genes may serve to protect against disease progression in the natural host. In addition to those described above, many genes were associated with leukocyte movement and adhesion (e.g., DEFA3 and KCNA3), and cell survival and growth (e.g., DNASE1L3, AXL, and DUSP5). A significant network linking functions to epidermal growth factor was also identified by Ingenuity Pathway Analysis (not shown). The network genes encoded sulfotransferases for glucosamine signaling (e.g., HS3ST2 and HS3T3A1), actin-binding proteins (e.g., FSCN2 and CDC42EP1), and proteins required for cytoskeleton and extracellular matrix structure (e.g., EMILIN1, TUBA1A, and COL17A1). These patterns indicate that AGMs may be able to better maintain basic cellular functions and organ structure, consistent with reports that lymph node architecture is better preserved during non-pathogenic infections [49].

### Species- and time-dependent gene expression changes in lymph nodes

We similarly examined the kinetics of gene expression changes within each species by using ANOVA to compare expression data by day post-infection, identifying 297 genes whose expression significantly changed in PT lymph nodes between day 10 and day 45+ (Figure S2A). Many genes induced at day 10 were linked to apoptosis and oxidative stress, with increased expression of DNA damage-inducible genes (e.g., SOD2, YWAE, GZMB, GZMH, BAK1, and PRF1), some of which are specifically associated with apoptosis of T lymphocytes. This supports the day-10 species ANOVA, showing early and high expression of genes associated with stress responses in pathogenic infection and confirms that these responses are specific to viral peak and abate in LNs thereafter. About one third of genes identified by the PT time-dependent ANOVA showed either no change or decreased expression at peak VL (day 10), followed by strong induction at day 45+. In general, these genes belonged to the functional categories of cellular assembly and tissue development. Of these genes (blue bar in Figure S2A), 41 showed high expression in AGMs as early as day 10, which persisted to day 45+ (Figure S2B). The expression of these genes may contribute to a successful antiviral response or to the maintenance of proper cell homeostasis in non-pathogenic hosts, but they are induced too late to be effective during pathogenic infection. Gene ontology analysis showed that the genes induced earlier in AGMs are necessary for growth, differentiation, and structure (e.g., RYR2, PCDHGA7, MYLPF, EEF2K, and CAMK2N2).

The lymph node AGM time ANOVA identified 170 genes whose expression changed significantly between day 10 and day 45+ post-infection, less than 30 of which were common with the LN PT time ANOVA (not shown). In contrast to what was observed in PT, most of these genes were highly induced in AGMs at day 10 but decreased by day 45+, and many encoded inflammatory mediators specifically associated with the interferon response (e.g., IFIT2, IFIT3, STAT1, IFITM2, IFITM3, CXCL10, and IFIH1). During this same time frame, high expression of these genes was sustained in PTs. These observations suggest that attenuation of interferon signaling at the transcriptional level may be an important characteristic of non-pathogenic SIV infection.

Collectively, analysis of LN revealed that most differential gene expression patterns in PTs vs. AGMs occurred at peak VL (day 10) and related to immune responses and cell death. During early pathogenic infection, species and time ANOVAs revealed high induction of genes associated with acute stress responses, DNA damage, and Th1 signaling. In AGMs, however, gene expression data suggested control of Th1 responses and maintenance of general cell homeostasis processes, including immune cell homeostasis. Though expression patterns in LNs of both species were more similar by day 45+, there was a clear tendency for sustained inflammatory responses during pathogenic infection, while IFN signaling was significantly attenuated by day 45+ during non-pathogenic infection.

### Gene expression patterns in peripheral blood

The time- and species-specific gene expression changes in peripheral blood were very similar to those observed in lymph nodes. Time ANOVAs in peripheral blood, comparing AGM expression levels at day 10 to day 45+, further confirmed evidence for an attenuated inflammatory response during non-pathogenic infection. Of 109 genes identified by AGM time ANOVA, one quarter were less induced or down-regulated by day 45+ (Table S5) and most of these were involved in IFN-signaling (e.g., MX1, MX2, OAS1, OAS2, CXCl10, IFI35, IFI27, and ISG20). Expression patterns identified by blood day 45+ species ANOVA (Figure S3) suggested the strong and sustained induction of genes associated with an inflammatory response only in PTs (Figure S3, blue bar; Table S6), many of which can be regulated by IFN (e.g., MX1, MX2, OAS1, and IFI27). One unique pattern identified by blood day-10 species ANOVA that was not observed in LNs, was a marked decrease, only in PTs, in expression levels of most genes at peak viral load (Table S7). Overall, this broad “suppressive” trend in PTs was seen in genes associated with the regulation of basic cellular homeostasis [50], including functions such as apoptosis regulation and the development of cells involved in the immune response. The decreased expression of these genes may in part be due to depletion of circulating CD4+ T cells in PTs at 10 days post-infection, either because of increased destruction, decreased production, or movement into tissues. Given the low proportion of CD4+ T cells in blood, however, it is unlikely that such depletion is responsible for all of these dramatic gene expression changes

### Species similarities of time-dependent gene expression signatures in colon

In contrast to LN and blood, we found that in the colon there were more genes that were significantly differentially expressed between time post-infection (day 10 vs. day 45+) than between species. The AGM time ANOVA identified 658 genes whose expression distinguished day 10 from day 45+. Most genes were unchanged or decreased at day 10 but were highly induced by day 45+, very similar to the PT time ANOVA pattern. In fact, nearly 300 genes from the colon AGM and PT time ANOVAs were in common (Figure S4). Functions most highly represented by these genes included tissue development, cell movement, growth and proliferation, death, and cell-cell signaling. These included genes encoding scaffolding proteins, muscle filaments, extracellular matrix components, and cell cycle/apoptosis regulators. Ingenuity Pathway Analysis was used to identify interactions between nearly 30 genes associated with TGFβ signaling and fibronectin (data not shown). These observations suggest that the expression of genes related to cell growth, proliferation, and apoptosis remain at baseline at peak VL and do not increase until late in acute infection. Collectively, the time ANOVA data suggest that the majority of SIV-induced transcriptional changes in the colon occur after peak VL in both species. Since most of the genes induced at viral set point were emblematic of broad tissue reconstruction, there appears to be a common response in the colon of both species that occurs in temporal relationship to the CD4+ T cell depletion found in each [40],[41],^51^.

**Figure 5:**
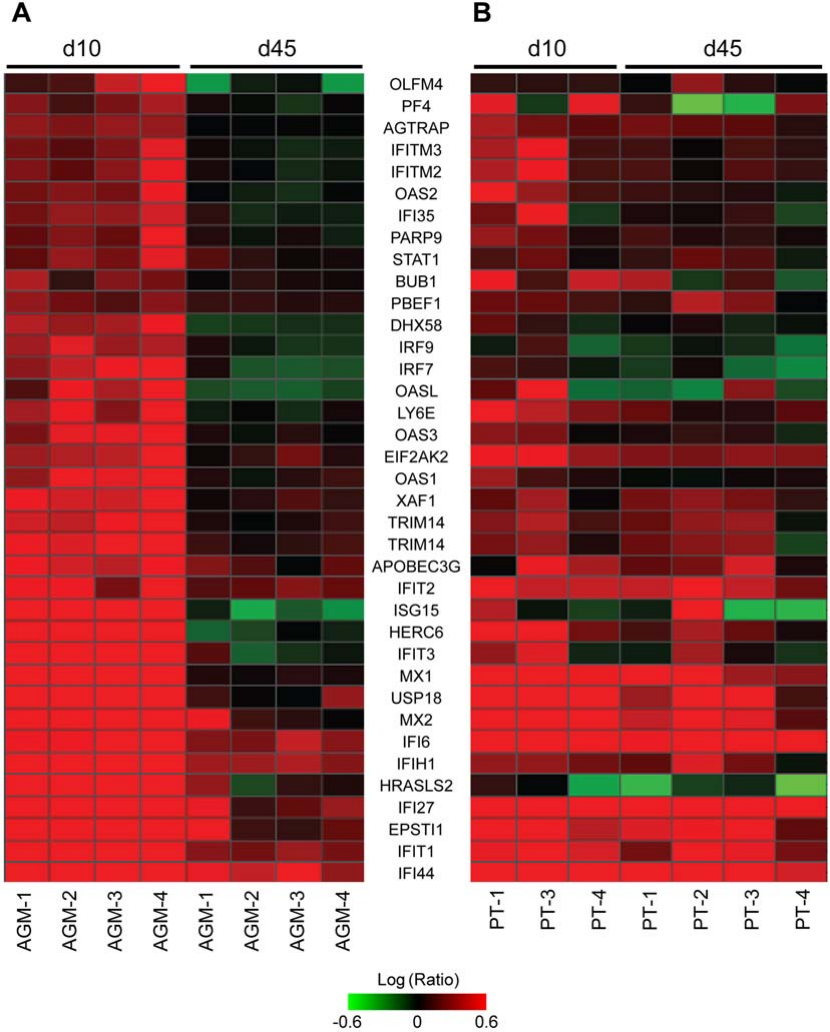
Gene expression changes in AGM colon between day 10 and day 45+. (A) Heatmap depicting 37 genes that were highly induced in AGMs at day 10 and significantly lower by day 45+. (B) Corresponding expression levels for PTs. Genes depicted were 2-fold regulated (*P*≤0.05) in at least 2 of 8 animals.

Of the nearly 700 genes identified by AGM time ANOVA in the colon, only a subset of 37 genes was induced at day 10 at high levels and significantly lower by day 45+. These genes are shown in Figure 5A, along with the corresponding expression in PTs (Figure 5B). Most are inflammatory mediators with many involved in type I interferon signaling, which is consistent with the patterns identified in the lymph nodes. Though expression levels consistently decreased in all AGMs, but not in PTs, from day 10 to day 45+, the expression levels of many of these inflammatory mediators were actually higher in AGMs than PTs at day 10. This suggests that AGMs not only show attenuation of an inflammatory response after acute infection, but that a more robust early inflammatory response in the colon may control viral replication and subsequent tissue damage. These results are consistent with a previous study that found an association between a stronger early IFN response and slower disease progression [52].

### Acute phase response signaling in the colon of pathogenic SIV model

**Figure 6:**
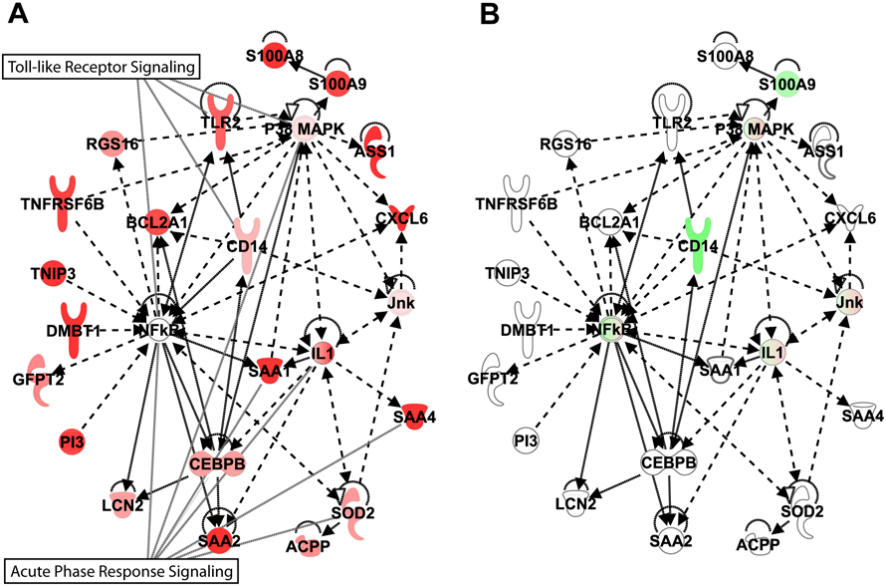
Expression of genes associated with acute phase response/NFkB signaling in colon of PT (A) and AGM (B). Ingenuity network analysis showing connection between genes associated with NFkB signaling overlaid with expression data from in-silico pooled samples of PT colon (A) or AGM colon (B) at day 10. Genes shown in red and green represent increased or decreased expression, respectively, relative to pre-infection levels.

Perhaps the most surprising finding was that the colon showed the smallest number of genes statistically distinguishing the two species at peak VL. This and the time ANOVAs suggest that the host transcriptional response to SIV infection in colon is similar in both species, and is consistent with the fact that CD4+ T cells are lost in each. Similar to what was observed in peripheral blood and LN, more gene expression changes differed during pathogenic vs. non-pathogenic infection in the colon at day 10 than at day 45+. Of 165 genes that distinguished PTs from AGMs in the day 10 colon ANOVA, 74 were highly induced only in the PTs (Table S8) Pathway analysis revealed that the top pathways/networks represented by these up-regulated genes were associated with the acute phase response. Figure 6 shows the most significant network linking these genes and centers to the transcriptional regulator, NFκB. Most genes in the network linked important acute phase response regulators such as p38MAPK, IL1, SOD2, and CEBPB, which in turn positively influence the induction of additional inflammatory mediators. The Toll-like receptor pathway was also prominent, with increased expression of TLR2 and CD14, possibly reflecting responses to bacterial products. This supports a report that TLR induction by bacterial products escaping the gut could trigger high expression of inflammatory mediators [27]. If so, it is evident that these pathways were not prominent in AGMs (Figure 6B) at this time, so it would seem unlikely that they were involved in the depletion of mucosal CD4+ T cells observed in this species.

The end of acute infection (day 45+) was associated with the expression of 106 genes in the colon that distinguished pathogenic and non-pathogenic infections (Figure 2C and Table S9). Here, tissue development and cell movement were identified as the top functional categories, followed by immune response, cell death, and proliferation. It has been reported that cell adhesion may play a role in maintaining the integrity of the mucosal barrier during pathogenic SIV infection [53] while dysregulation of growth factors and epithelial repair may compromise barrier integrity [28],[54],[55]. Therefore, it was interesting that most of the genes in the first category associated with membrane development, extracellular matrix, and cell adhesion (RPL37A, CEACAM1, CEACAM7, COL4A2, ALOX12, DSC2, WNT3, and MAL) were either highly induced only in AGMs or showed significantly decreased expression specifically in PTs. Notably, up-regulation of structural genes in this species ANOVA was negligible in PTs. Although both species appeared to initiate programs involving structural integrity genes by day 45+ (as seen in the time ANOVAs, Figure S4), subtle changes specific to AGMs may be part of a mechanism associated with the absence of disease progression. Evidence for bacterial translocation was again observed at day 45+ by induction of TLR 4 expression in PTs. Also, consistent with the observations in LN and peripheral blood, genes encoding inflammatory mediators were more highly induced at day 45+ in PT than in AGM colon.

In sum, most gene expression patterns in the colon were common to both the PT and the AGM, and between the peak of viremia (day 10) and day 45+ post-infection, animals of each species revealed significant increases in the expression of genes responsible for cell growth and development. Species-specific differences revealed a strong acute phase response in PTs at peak VL (day 10) while induction of structural genes was most prominent in AGMs at viral set point (day 45+). Additionally, though the inflammatory response was high and sustained in PTs, there was a higher IFN response in AGM colon at day 10, which was significantly attenuated by day 45+ post-infection.

## Discussion

This study used two NHP species to comprehensively analyze the host response associated with non-pathogenic and pathogenic SIV infection during the acute phase. Our primary findings are summarized in Table 1. Most species-specific gene expression differences occurred in LN at the time of peak VL, and the majority of time-dependent changes for a given species were in the colon between day 10 and day 45+. Overall, gene expression patterns in PTs (pathogenic infection) suggest a strong and sustained immune activation, including CTL activity and Th1 profiles. In contrast, the intensity and kinetics of the inflammatory response in AGMs (non-pathogenic infection) suggest strong interferon signaling at day 10, which was then attenuated by day 45+. The AGMs also exhibited fewer gene expression changes related to cell homeostasis (defined here as genes associated with basic cellular functions, such as metabolism, growth, and continued hematopoietic regeneration).

**Table 1:** Summary of Global Transcriptional Patterns during Pathogenic (PT) and Non-Pathogenic (AGM) SIV Infection.

Lymph Node		
	AGM	PT
**D10**	↑ control of Th1 response	↑ Th1 response: CTL signaling, DC maturation, IFNγ signaling
	↑ IFNα signaling	↑ oxidative stress, DNA damage
	↑ cell assembly	↑ anti-apoptotic mediators
	Maintenance of progenitor cell regeneration	↑ immune activation
**D45+**	Attenuation of IFN signaling	Sustained IFN response
		Delayed induction of tissue development

Blood		
	AGM	PT
**D10**	No change in cell growth	↓ basic cell homeostasis
**D45+**	Attenuation of IFN signaling	Broad inflammatory response
		DNA damage, oxidative stress

Colon		
	AGM	PT
**D10**	↓ cell growth, proliferation and development	↓ cell growth, proliferation and development
	Higher Type I IFN response	↑ Inflammation and damage
		↑ Acute phase response: NFkB signaling, complement, TLR signaling
**D45+**	↑ tissue reconstruction	↑ tissue reconstruction
	Attenuation of IFN signaling	Sustained inflammation

Shown are the most statistically significant patterns suggested by the gene expression data at peak VL (day 10) and post-peak (day 45+) in LN, blood and colon of SIV-infected animals.

Although our study is the first to use global gene expression profiling to compare the host response during pathogenic and non-pathogenic SIV infection, a number of previous studies have also used NHP models in which infection outcome differs in an attempt to elucidate mechanisms associated with disease progression. Several themes have begun to emerge. For example, Cumont et al [56] also observed a link between dysregulation of immune activation and SIV pathogenesis, finding high levels of immune activation in LN T cell zones of SIV-infected macaques, but low levels of T cell activation and high levels of B cell activation in AGMs. This is consistent with our gene expression data. Early induction of T cell signaling genes during pathogenic infection supported a trend for Th1 cell differentiation and proliferation, cytotoxic T cell activity, and extensive IFNγ signaling. Increased IFNγ expression, an indicator of the Th1 response, has been detected in lymphoid tissue of macaques [24],[43],[57], and dysregulation of Th1 agonists may contribute to pathogenesis [58]. In contrast, prominent Th2 cytokine profiles have been associated with SIV infection during non-pathogenic infection [59].

Levels of CD8+ T cell activation have been directly correlated with accelerated AIDS progression [16]–[18]. In our study, Ki67 levels were statistically higher in PT CD8+ T cells than in AGMs, and these levels correlated well with gene expression profiles indicative of a more pronounced CTL response in PTs (e.g., up-regulation of IFNγ, immunoproteasomes, and granzymes). In contrast, CD8+ T cell Ki67 expression and transcriptional profiles in the AGM indicated lower levels of T cell activation and less prominent Th1 patterns [34], [60]–[62]. Although similar observations have been attributed to a low immune response of AGMs to core SIVagm protein [63], we found that SIV-specific antigen responses were similar in both species [39]. This supports studies finding comparable SIV-specific cellular responses in both pathogenic and non-pathogenic models of SIV infection [64]–[67]. Therefore, the increased expression of genes associated with CTLs during pathogenic infection may be a result of improperly-regulated activation of bystander immune cells [34].

Loss of T regulatory cells (Tregs) [68] and cell cycle dysregulation [69] have been associated with pathogenic SIV-infection. For example, microarray analyses of macaque LN and jejunal biopsies [53],[57] have shown dysregulation of cell cycle and increased induction of cytolytic activity as part of a localized host response. We also observed the acute loss of Tregs during pathogenic infection and cell cycle dysregulation in the transcriptional analysis of LNs at peak VL. Additionally, high expression levels of FOXO3, which negatively regulates T cell activation and autoinflammation [70], were unique to AGM blood (data not shown). Taken together, this suggests that failure to control T cell activation/proliferation may contribute to poor outcome [56],[62].

Numerous studies have demonstrated that varied levels of apoptosis, or the induction of apoptosis-related genes, are linked to species-specific differences in disease susceptibility [34],[56],[71]. Global gene expression patterns for apoptotic mediators formed a complex picture in our analysis. Cell death genes made up the most significant functional group that distinguished PT from AGMs at day 10 in LN. However, the number of induced genes associated with death receptor signaling were nearly equal in AGMs and PTs, while the number of genes associated with protection from apoptosis were higher in PTs. Higher levels of annexin staining [39], loss of CD4+ T cell counts, and the induction of oxidative stress and DNA damage-inducible genes in PT lymph node and blood suggest an increased apoptotic response in PTs. The anti-apoptotic gene expression pattern in the LN species ANOVA may point to a negative feedback mechanism in PTs that attempts to protect the host from excessive cell death. However, because apoptosis is not generally a transcriptional event, it is difficult to interpret such gene expression changes and to directly correlate them with apoptotic events, except as they relate to increased expression of survival factors.

The increased expression of genes associated with cell damage and death in PTs may be due in part to high and sustained levels of inflammation, and several studies have associated such a response with SIV/HIV pathogenicity [24],[28],[53],[58]. However, it has been unclear whether there is an immediate anti-inflammatory environment in AGMs, or whether these animals are even capable of mounting a Type I IFN response to SIV. Our results demonstrate that acute SIV infection does indeed activate Type 1 IFN signaling in AGMs. For example, a group of eight IFNα genes were induced up to 10 fold over pre-infection levels in AGM LN at peak VL. We have previously shown that IFN can block SIV replication at early stages of infection in vitro [72], and our current analysis indicates that early Type I and II IFN responses may help AGMs to limit SIV pathogenesis.

Thus, AGMs and PTs both appeared to mount an early antiviral response, although the response of AGMs was stronger, earlier, and perhaps more effective. By day 45, the IFN response was significantly attenuated in AGMs, but less so in PTs. It is possible that IFN signaling was actively suppressed in AGMs shortly after peak VL, thereby limiting the induction of inflammatory mediators that would otherwise hasten disease progression. Alternatively, the lower induction of IFN genes may be a consequence of lower viral loads in the LN of these animals, although the same pattern of attenuation was observed in colon, where viral loads were similar to those observed in PTs.

The strong and early IFN signaling in AGM colon is consistent with reports suggesting that a strong innate immune response in mucosal tissue is a mechanism for limiting SIV pathogenesis [52],[53],[73]. However, it is also notable that many of the time-dependent gene expression changes in the colon were common to both species and were associated with the regulation of structural genes. This finding indicates that normal cell homeostasis was disrupted in the context of both pathogenic and non-pathogenic infection at the primary site of SIV infection [74]. Although the initial inflammatory environment could lead to damage of the gut mucosa in both PTs and AGMs [27], the early attenuation of the response in AGMs may help to reduce such damage. In contrast, the loss of mucosal integrity in PTs may contribute to pathogenesis [28],[75],[76].

In LN, genes encoding growth or hematopoietic factors and gamma-delta T cell differentiation were significantly more induced in AGMs than in PTs. Genes encoding proteins implicated in B cell homing and differentiation, as well as the development and chemotaxis of mast cells and eosinophils, were also induced in AGMs. Although this could be due to changes in lymphoid cell distribution, it is also consistent with the hypothesis that certain cell subpopulations in the AGM immune system are better adapted to control SIV infection than their counterparts in the PT. Superior immune function may also be linked to the unique induction of cell survival and structural genes observed in AGM LN, since the destruction of LN architecture is associated with rapid disease progression in SIV-infected macaques [49]. Although we did not observe LN destruction at the relatively early time points studied in this experiment, immunological data showed that PTs had higher levels of immune activation and apoptosis in lymphoid tissues than did AGMs. AGM-specific induction of structural genes during early acute infection may also lead to better preservation of LN architecture at later stages of infection. Therefore, our gene expression data suggest that AGMs may be able to maintain overall immune homeostasis through LN preservation, chemotaxis of certain immune cell subsets, and the general ability to maintain normal cellular processes despite continued viral replication.

Global gene expression profiling is increasingly being used to study lentiviral infection of cell lines, NHPs, and human patients. Similar to the findings reported here, many of these studies have also observed differences in the regulation of genes related to apoptosis and the immune response, including those associated with IFN signaling, disruption of immune cell homeostasis, and the preservation of LN architecture [47], [57], [77]–[81]. Most intriguing are similarities in the expression patterns we observed in SIV-infected AGMs to those identified in lymphoid tissue of HIV patients on HAART [82]. In both cases, genes associated with cytotoxic T cells showed a decrease in expression compared to that observed in SIV-infected PTs or untreated HIV-infected humans. In contrast, signs of immune activation are attenuated in patients on HAART, as we observed in AGMs. Genes that contribute to extracellular matrix structure and that support hematopoietic cell growth are also induced in patients on HAART. These striking similarities suggest that mechanisms independent of viral suppression—possibly the quality of immune activation or resolution—are different in the context of pathogenic vs. non-pathogenic infection. These findings also underscore the relevance and reproducibility of genomic SIV studies for elucidating mechanisms of AIDS pathogenesis.

In summary, our findings emphasize the importance of tissue compartmentalization and kinetics in SIV pathogenesis as well as the unifying pattern of IFN induction and attenuation in the natural host. Because the greatest number of gene expression changes distinguishing pathogenic from non-pathogenic infection occurred in lymph node at peak viral load, we propose that this may be a promising site and time point for further study. To our knowledge, this is the first time that such a comprehensive data set—derived using the macaque genome to directly compare gene expression profiles during acute stages of pathogenic and non-pathogenic SIV infection—has been made available to the public.

## Materials and Methods

### Virus

The original SIVagm.sab92018 primary isolate [6] was kindly provided by Dr. O. Diop at the Pasteur Institute in Dakar, Senegal and is CCR5- and CXCR4-duo-tropic [83]. Our viral stock was obtained by infecting one SIV negative C. sabaeus (Caribbean origin from the colony at the Center for Primate Neuroethology and Neuropsychiatric Institute, University of California, Los Angeles) with 300 50% tissue culture infectious doses (TCID_50_) of SIVagm.Sab92018 (Dakar), and by collecting plasma at day +10 following IV inoculation. The virus stock (pure plasma) was titrated on SupT1 cells at a titer of 1250 TCID_50_/ml, corresponding to 1.06×10^9^ RNA copies/ml (as measured by qRT-PCR; see below) and 7 ng/ml p27 (as measured by p27 ELISA, Zeptometrix, Buffalo, NY) as described in ^2^ and in Methods.

### SIVagm infection of NHPs and tissue collection

Ethics Statement: All animal and *in vitro* procedures were performed using standard protocols and according to guidelines approved by the University of Washington Environmental Health and Safety Committee, the Occupational Health Administration, the Primate Center Research Review Committee, and the Institutional Animal Care and Use Committee.

The four adult male AGMs (10 years old, weighing 6.0 kg, interquartile range 5.8–6.2) and four adult male PTs (11 years old, weighing 16 kg, IQR 15.3–17.0) included in this study were housed at the Washington National Primate Research Center (WaNPRC). At day 0, animals were inoculated intravenously with 600 50% tissue culture infectious doses (TCID_50_) of SIVagmSab92018 and followed until necropsy at day 45 or day 49 (subsequently noted as day 45+). Blood samples were collected at days −14, 10, and 45+. At days −14, 10, and 45+, biopsies were obtained from the colon and lymph nodes (inguinal).

### RNA extraction

For microarray and VLs on tissues stored in RNAlater (colon and lymph node biopsies) or in OCT (lymph node biopsies), tissues were homogenized using a Polytron PT2100 tissue grinder (Kinematica, Switzerland) and RNA was extracted using TRIZOL Reagent according to manufacturer's instructions (Invitrogen). Whole blood was collected into PAXgene tubes for RNA extraction with the PAXgene RNA blood kit (Qiagen, Valencia, CA). RNA concentrations were quantified on an ND-1000 UV-Vis spectophotometer (Nanodrop, Wilmington, DE) and controlled for integrity and purity on a capillary electrophoresis system (Agilent 2100 Bioanalyzer; Agilent Technologies, Santa Clara, CA) and processed for microarray as described (Kash et al., 2006a; Kash et al., 2006b; Kobasa et al., 2007)

### Viral load measurement

RNA VLs in plasma and tissues were measured by real-time quantitative PCR (ABI PRISM 7700, Applied Biosystem Foster City, CA) in a one step RT-PCR reaction (HoTaq One Step-RT PCR Mix; Molecular Cloning Laboratories, South San Francisco, CA), using primers and probes as previously described (Diop et al., 2000).

### Expression microarray

Gene expression profiling on whole blood, colon, and LN biopsies at days 10 and 45+ was compared to the baseline time point (day −14) for each tissue and each animal. Probe labeling was performed using Low RNA Input Fluorescent Linear Amplification kit (Agilent Technologies, Santa Clara, CA). Slide hybridizations were performed with rhesus macaque (*Macaca mulatta*) oligonucleotide microarrays containing 18,000 rhesus probes (Agilent), representing over 17,000 unique rhesus genes. The platform used here was based on the 3′-UTR sequences of rhesus transcripts, as identified in the 6×-coverage rhesus genome (MMUL1.1). Based on available sequences, the probes of the rhesus array have 98% sequence identity to corresponding sequences in *M. nemestrina* or *M. fascicularis* [84]. Extending this to *C. sabaeus*, we estimate this to also have better than 97% identity to *M. mulatta*.

Each microarray experiment was done with two technical replicates by reversing dye hybridization for experimental and reference samples. Slides were scanned with an Agilent DNA microarray scanner, and image data were processed using Agilent Feature Extractor Software (Agilent Technologies, Palo Alto, CA), which also performed error modeling. All data were subsequently uploaded into Rosetta Resolver 7.0 (Rosetta Biosoftware, Kirkland, WA) and Spotfire Decision Suite 8.1 (Spotfire, Somerville, MA) for data analysis. In accordance with proposed MIAME (minimum information about a microarray experiment) standards [85], all data described in this report, including sample information, intensity measurements, gene lists, error analysis, microarray content, and slide hybridization conditions, are publically available at http://viromics.washington.edu.

The Resolver system performs a squeeze operation that creates ratio profiles by combining replicates while applying error weighting. The error weighting consists of adjusting for additive and multiplicative noise. A *P* value is generated that represents the probability that a gene is differentially expressed. The Resolver system then combines ratio profiles to create ratio experiments using an error-weighted average as described in Roland Stoughton and Hongyue Dai, Statistical Combining of Cell Expression Profiles (US Patent #6,351,712, February 26, 2002). For each microarray experiment, the calculation of mean ratios between expression levels of each gene in the analyzed sample pair, standard deviations, and *P* values was performed using Resolver.

In this study, genes selected for data analysis had a greater than 95% probability of being differentially expressed (*P*≤0.05) and fold change ≥2. Ingenuity Pathway Analysis (IPA) software and Entrez Gene (www.ncbi.nlm.nih.gov/sites) were used for gene ontology analysis. Analysis of Variance (ANOVA), a factorial-based analysis method for determining statistical differences between means of different populations, was used to identify genes that statistically distinguished either species or time points.

### Cell counts, phenotype, and functional analysis by flow cytometry

Complete blood counts (CBC) were determined at the WaNPRC Clinical Lab in Seattle as well as by absolute counting on 50 µl whole blood, using Trucount absolute counting tubes (BD Biosciences, San Jose, CA). Phenotyping was performed by cell surface staining. FACS data were analyzed using FlowJo software with standard gating strategies and then transferred into analysis and graphic software (Excel, StatView (Abacus Concepts, Berkeley, CA)). Multifunctional cytokine analysis was performed after stringent gating of each cytokine positive population and subsequent Boolean gating (FlowJo).

## Supporting Information

Figure S1 Viral loads and CD4 counts in PBMC, colon, LN. A. Cellular viral loads expressed as number of RNA copies of SIVagmSab92018 per µg of total cell-associated RNA. Each animal is represented by a different color and symbol. B. Frequency of CD4+ T cells among total CD3+ T cells, expressed as percent CD4+.
(0.65 MB TIF)

Figure S2 Time ANOVA in PT lymph node. A. 297 genes from one-way time ANOVA in PT lymph node distinguished day 10 from day 45+. The blue bar indicates genes that were induced at day 45+ in PTs. B. Expression levels in AGMs and primary sequence names for 41 genes from the blue bar selection. Each column represents gene expression data from an individual animal. A full view is shown with green and red colors showing decreased or increased levels of mRNA expression relative to d-14. Only genes with ≥2-fold change (P≤0.05) in at least 2 of 8 experiments are shown. 2-D hierarchical clustering was performed using Resolver System software with agglomerative algorithm, average link heuristic criteria, and Cosine correlation metric.
(0.90 MB TIF)

Figure S3 Gene expression profiles distinguishing AGM from PT in blood. 254 genes were identified by day 45+ species ANOVA and are shown with cutoffs and clustering as described in Figure S2. The blue bar indicates 63 genes that were only induced in PTs at day 45+ and that are listed in Table S6.
(0.81 MB TIF)

Figure S4 Differences in gene expression profiles based on time post-infection in AGM and PT colon. One-way time ANOVAs in AGM and PT colon distinguished day 10 from day 45+. 294 genes were common to time ANOVAs from both species. A. Expression pattern for 294 common time ANOVA genes in AGMs. Cutoffs and color schemes are as described in Figure S1. B. The corresponding expression levels for these genes are also shown in PTs.
(0.42 MB TIF)

Table S1 Cell death genes
(0.02 MB XLS)

Table S2 Immune response genes
(0.03 MB XLS)

Table S3 Genes uniquely induced in PTs
(0.03 MB XLS)

Table S4 Genes uniquely induced in AGMs
(0.03 MB XLS)

Table S5 Genes less-induced at d45+ in AGM blood
(0.02 MB XLS)

Table S6 Genes up-regulated at d45+ in PT blood
(0.02 MB XLS)

Table S7 Genes down-regulated at day 10 in PTs
(0.05 MB XLS)

Table S8 Genes up-regulated in PTs from d10 colon species ANOVA
(0.02 MB XLS)

Table S9 Colon d45 species ANOVA
(0.03 MB XLS)
