# Supplementary figures and images for: Transcriptional Profiling in Pathogenic and Non-Pathogenic SIV Infections Reveals Significant Distinctions in Kinetics and Tissue Compartmentalization
Source: PLoS Pathog. 2009 Feb 13;5(2):e1000296. doi: 10.1371/journal.ppat.1000296 (PMC2633618; doi:10.1371/journal.ppat.1000296)

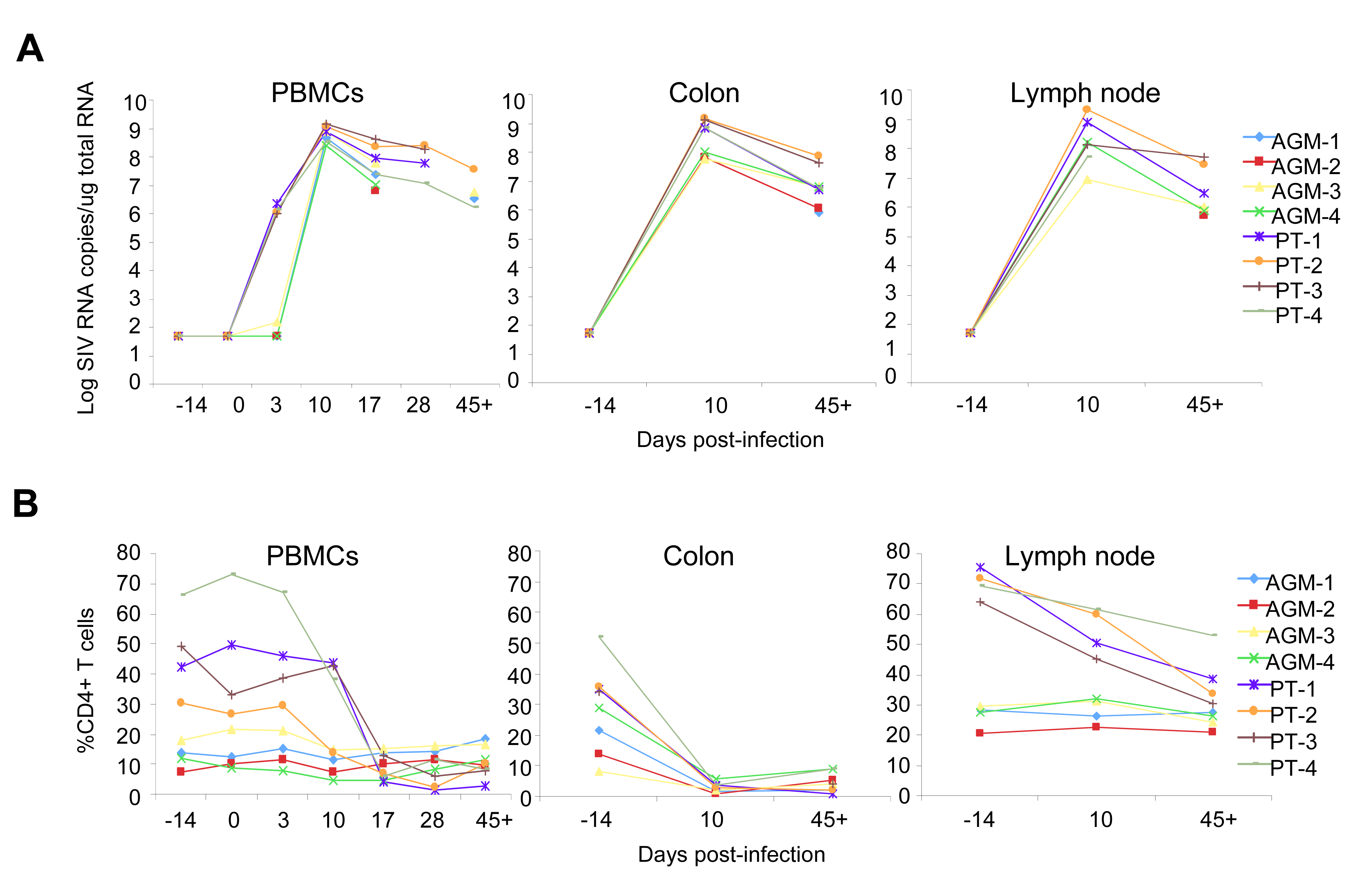

Supplement: Figure S1 — Viral loads and CD4 counts in PBMC, colon, LN. A. Cellular viral loads expressed as number of RNA copies of SIVagmSab92018 per µg of total cell-associated RNA. Each animal is represented by a different color and symbol. B. Frequency of CD4+ T cells among total CD3+ T cells, expressed as percent CD4+. (0.65 MB TIF) [file ppat.1000296.s001.tif]

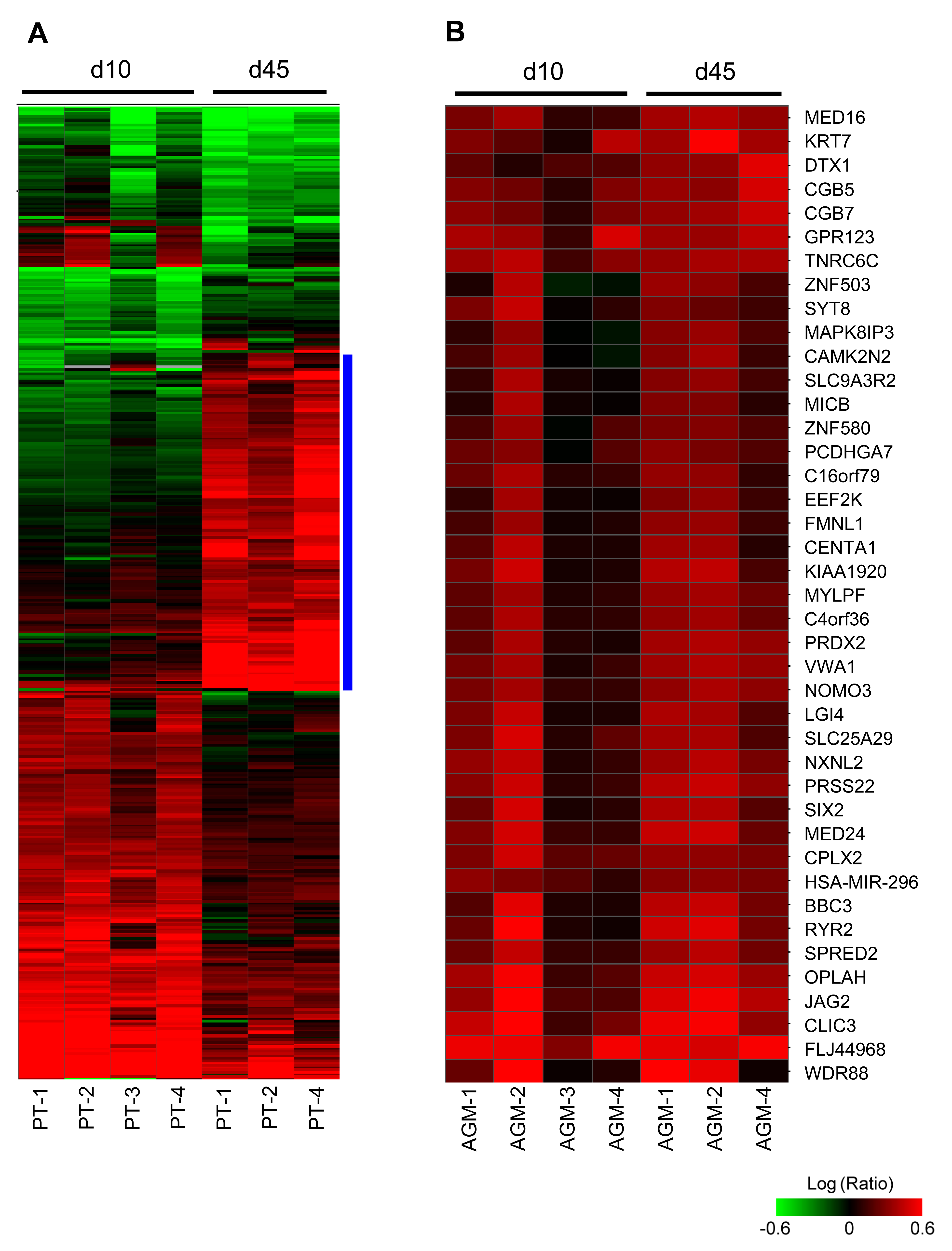

Supplement: Figure S2 — Time ANOVA in PT lymph node. A. 297 genes from one-way time ANOVA in PT lymph node distinguished day 10 from day 45+. The blue bar indicates genes that were induced at day 45+ in PTs. B. Expression levels in AGMs and primary sequence names for 41 genes from the blue bar selection. Each column represents gene expression data from an individual animal. A full view is shown with green and red colors showing decreased or increased levels of mRNA expression relative to d-14. Only genes with ≥2-fold change (P≤0.05) in at least 2 of 8 experiments are shown. 2-D hierarchical clustering was performed using Resolver System software with agglomerative algorithm, average link heuristic criteria, and Cosine correlation metric. (0.90 MB TIF) [file ppat.1000296.s002.tif]

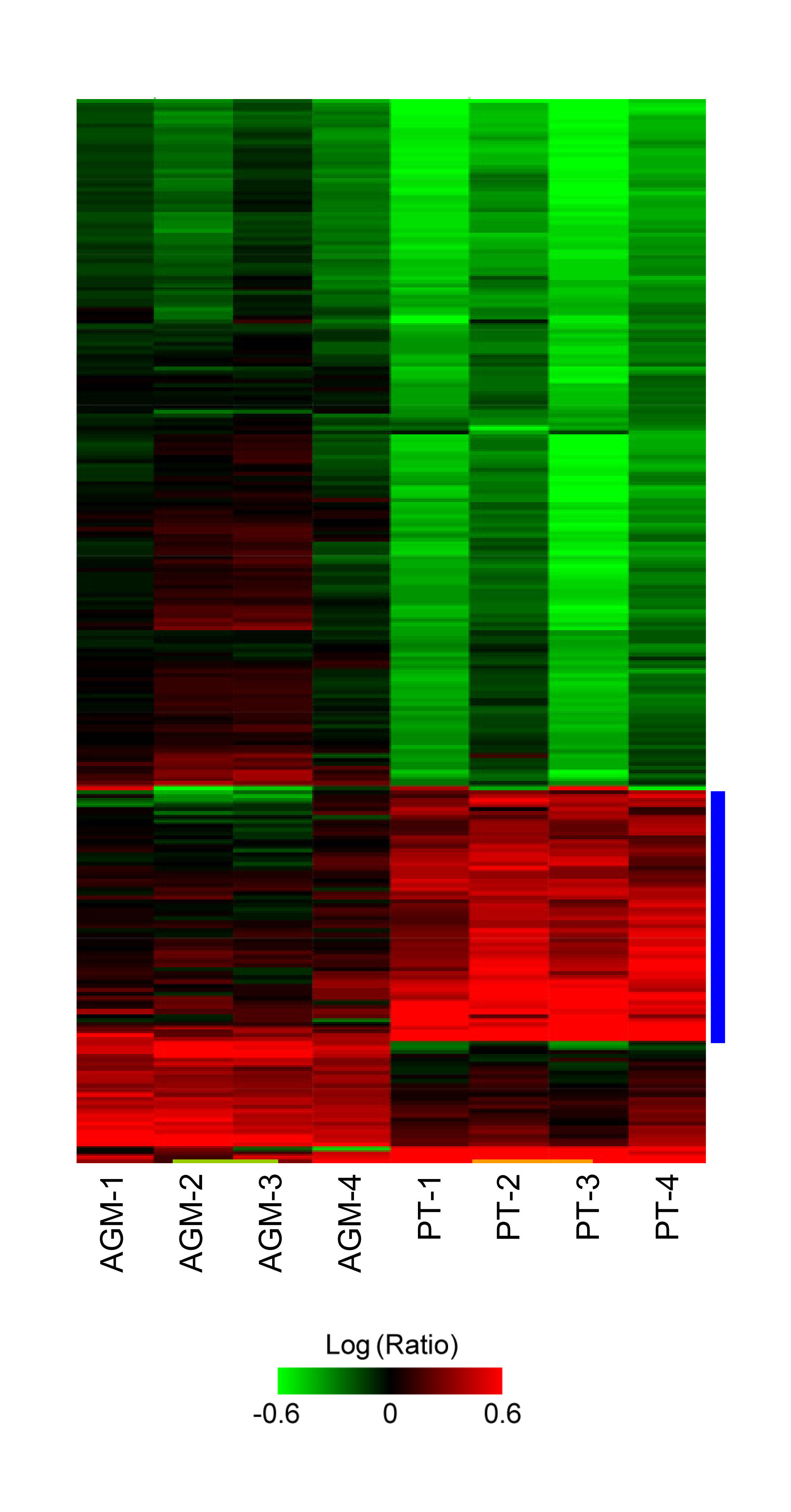

Supplement: Figure S3 — Gene expression profiles distinguishing AGM from PT in blood. 254 genes were identified by day 45+ species ANOVA and are shown with cutoffs and clustering as described in Figure S2. The blue bar indicates 63 genes that were only induced in PTs at day 45+ and that are listed in Table S6. (0.81 MB TIF) [file ppat.1000296.s003.tif]

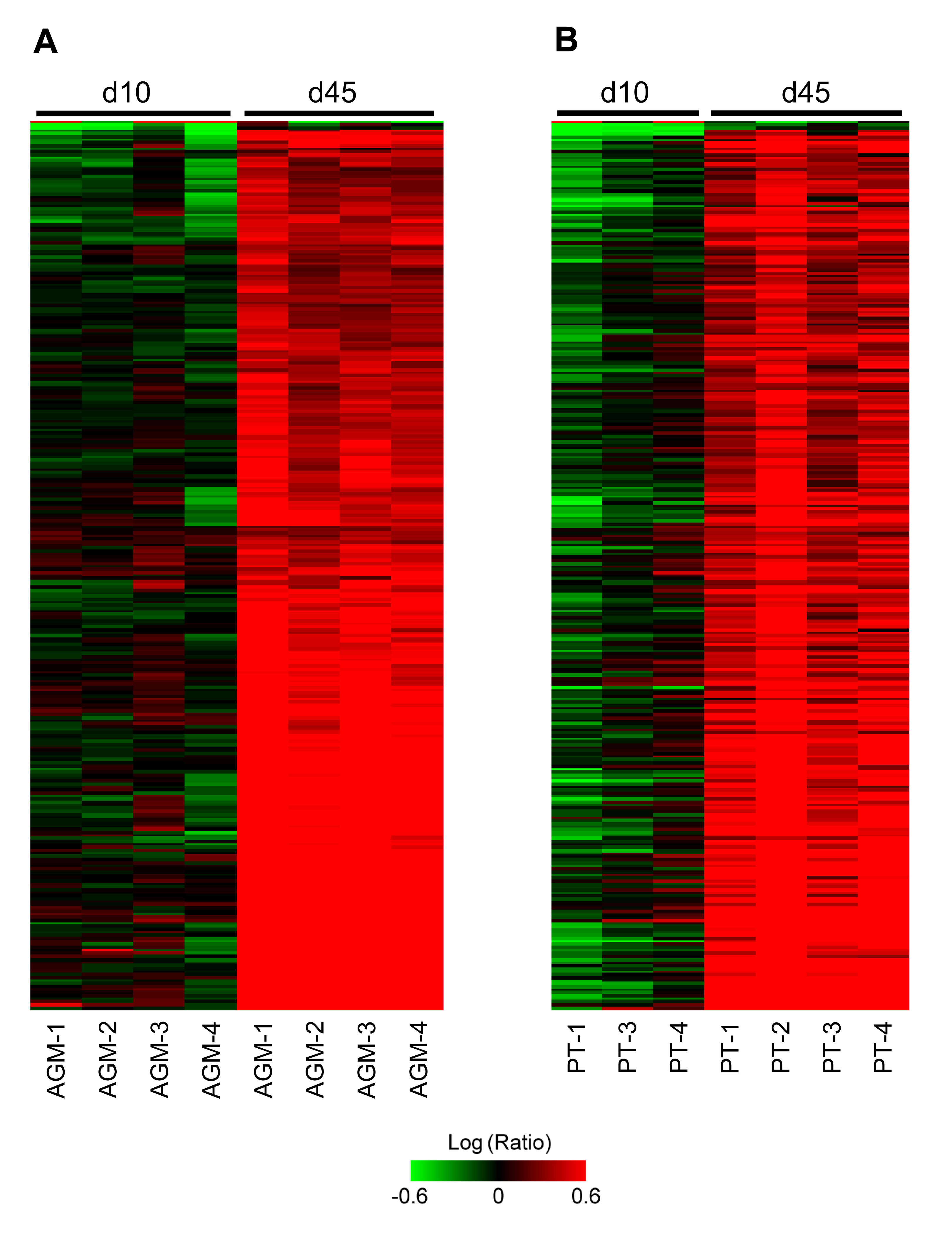

Supplement: Figure S4 — Differences in gene expression profiles based on time post-infection in AGM and PT colon. One-way time ANOVAs in AGM and PT colon distinguished day 10 from day 45+. 294 genes were common to time ANOVAs from both species. A. Expression pattern for 294 common time ANOVA genes in AGMs. Cutoffs and color schemes are as described in Figure S1. B. The corresponding expression levels for these genes are also shown in PTs. (0.42 MB TIF) [file ppat.1000296.s004.tif]
